# A Rare Presentation of Non-systemic Vasculitic Neuropathy Mimicking Guillain-Barré Syndrome: A Case Report

**DOI:** 10.7759/cureus.54945

**Published:** 2024-02-26

**Authors:** Chathuri L Munagama, Wasundara S Wathurapatha, Varithamby T Rajendiran, Shehan Silva

**Affiliations:** 1 University Medical Unit, Colombo South Teaching Hospital, Colombo, LKA; 2 Neurology, Colombo South Teaching Hospital, Colombo, LKA; 3 Medicine, Colombo South Teaching Hospital, Colombo, LKA; 4 Medicine, Faculty of Medical Sciences, University of Sri Jayewardenepura, Boralesgamuwa, LKA

**Keywords:** guillain-barré syndrome, painful sensory motor peripheral neuropathy, purpuric rash, asymmetrical polyneuropathy, vasculitic neuropathy

## Abstract

Vasculitic neuropathy typically presents as a painful, asymmetrical sensory-motor polyneuropathy, more commonly demonstrating a mononeuritis multiplex. We present the case of a 63-year-old woman who experienced acute-onset flaccid weakness in all four limbs following an episode of diarrhea. Guillain-Barré syndrome (GBS) was considered, which supported acute motor axonal neuropathy (AMAN) in the nerve conduction study (NCS). On the second day of treatment with intravenous immunoglobulin (IVIG), a vasculitic-type rash appeared along with limb pain. Furthermore, the asymmetrical sensory and motor weakness did not respond well to the treatment. A positive skin biopsy, however, with a negative nerve biopsy combined with repeat NCSs demonstrating mononeuritis multiplex, confirmed the diagnosis of non-systemic vasculitic neuropathy (NSVN) based upon Brighton Case Collaboration type 3. This presentation underlines the significance of considering vasculitic neuropathy as a potential diagnosis and highlights the importance of an accurate diagnosis, as this condition can be effectively treated.

## Introduction

Non-systemic vasculitic neuropathy (NSVN) is a frequently overlooked condition within the spectrum of vasculitic neuropathies. It is a rare, recognized condition characterized by necrotizing inflammation of the vessels, causing luminal narrowing of the vasa nervorum, and leading to ischemic injury to the peripheral nerves. It was first defined in 1987 [1]. The mean age of onset is 60.0 (+/-14.8) years, with a relatively even distribution between the sexes [2]. Most vasculitides (85%) are systemic and involve multiple organs and tissues in the body, whereas only 10%-15% of patients present with NSVN [3]. Non-systemic vasculitic neuropathy is typically an acute, relapsing neuropathy. However, it can have a slow progressive course. It is typically characterized by an asymmetrical, painful presentation of sensory or sensory-motor peripheral neuropathy, which is more predominant distally [2].

Three distinct patterns of NSVN have been identified: multifocal neuropathy, distal symmetrical polyneuropathy, and asymmetrical polyneuropathy [4]. While the gold standard for diagnosis is a peripheral nerve biopsy, the Brighton Collaboration case definition recognizes three levels of vasculitic neuropathy, taking into account the reduced sensitivity of the biopsy [5]. Additionally, NSVN encompasses subtypes and variants such as Wartenberg's migratory sensory neuropathy, post-surgical inflammatory neuropathy, and cutaneous polyarteritis nodosa [2].

Optimal management of NSVN involves dual therapy with corticosteroids and steroid-sparing agents, as evidence suggests that this approach yields superior outcomes compared to corticosteroid monotherapy while minimizing the risk of systemic organ involvement [6].

## Case presentation

A 63-year-old woman presented with generalized abdominal pain and delayed bowel opening for two days. There was no fever or vomiting. Plain radiography of the abdomen revealed fecal loading. A contrast-enhanced CT (CECT) of the abdomen showed proximal small bowel volvulus with multiple enlarged lymph nodes and no features of bowel ischemia. The patient underwent a diagnostic laparoscopy, which showed multiple small bowel adhesions that were attributed to the hysterectomy done for leiomyoma. On the following day, she developed severe diarrhea (eight to 10 times per day) in the absence of fever or vomiting. On the fifth day of diarrhea, the patient developed acute-onset, gradually progressive asymmetrical upper and lower limb weakness and numbness. There was no bladder, bowel, or cranial nerve involvement. She did not have any symptoms suggestive of a solid malignancy or a hematological malignancy. The patient denied significant medical history or family history and was not on any long-term medications, including native treatment. There was no significant high-risk sexual behavior associated with contact with a human immunodeficiency virus infection or occupational or environmental exposure to toxins.

On examination, the patient had flaccid quadriparesis and bilateral foot drop. The distal motor power was 1/5 in the lower limb and 2/5 in the upper limb, and the proximal motor power was 2/5 in the lower limbs and 3/5 in the upper limbs, with the right side more affected than the left. All limb reflexes and modalities of sensation like pain, light touch, and proprioception were reduced asymmetrically in the four limbs distally. She did not have autonomic dysfunction, including orthostatic hypotension. The rest of the system examination findings were normal.

**Figure 1:**
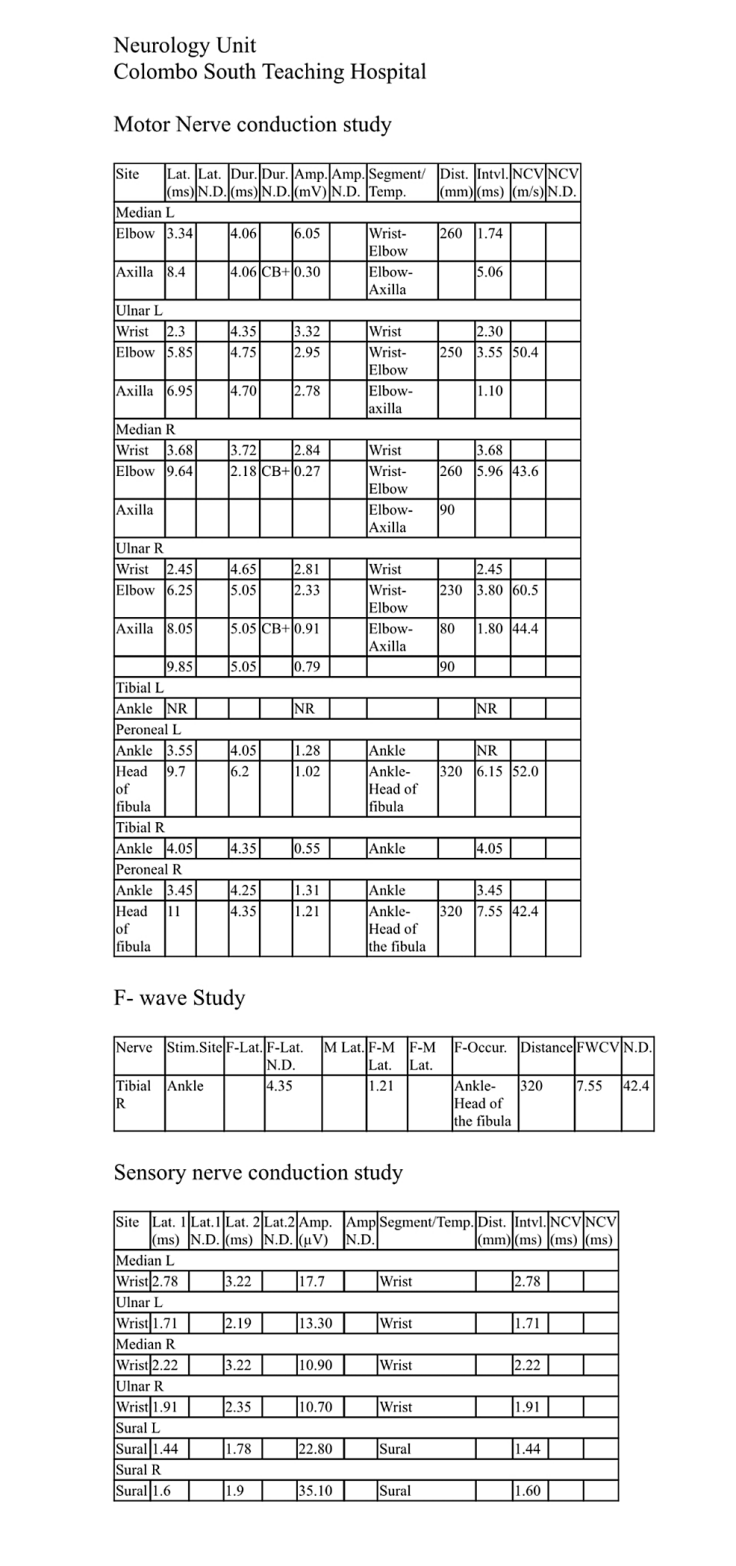
The first nerve conduction study Lat: latency; Dur: duration; Amp: amplitude; Dist: distance; Intvl: interval; NCV: nerve conduction velocity; ND: normal duration; CB+: conduction block; L: left; R: right; NR: normal range; Temp: temperature; Stim: stimulus; F-Lat: F-wave latency; M-Lat: M-wave latency; F-Occur: F-wave occurrence; FWCV: F-wave conduction velocity

**Figure 2:**
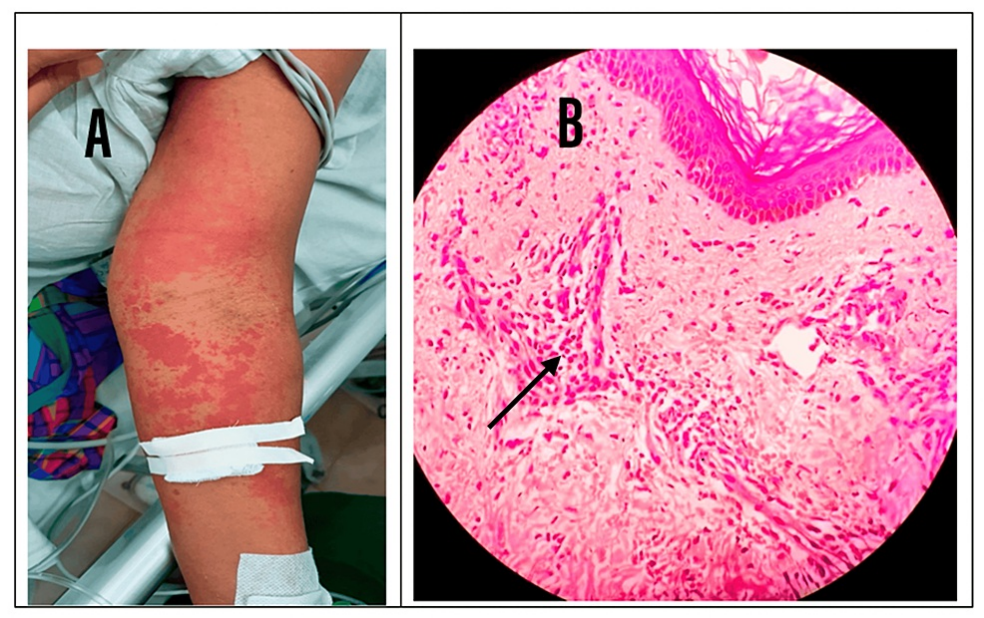
(A) Vasculitic-type skin rash; (B) Leukocytoclastic vasculitis demonstrated with an arrow in the skin biopsy

The initial nerve conduction study (NCS) done on the third day of the weakness demonstrated acute motor axonal neuropathy (AMAN). Guillain-Barré syndrome (GBS) was considered the diagnosis with the NCS and the initial clinical picture (Figure 1). Intravenous immunoglobulin (IVIG) 0.4 g/kg/day was administered for five days. On the second day of IVIG administration, she developed a centrifugal palpable, non-blanching, erythematous skin rash involving limbs with no involvement of the face (Figure 2A). Furthermore, there was severe neuropathic pain in all four limbs. Other laboratory investigations and imaging were done for further evaluation (Table 1).

**Table 1:** Laboratory investigations and imaging PT: prothrombin time; INR: international normalized ratio; ANCA: antineutrophil cytoplasmic antibodies; 2D: two-dimensional

Investigation	Value	Reference range
White cell count	8,370	4,000-11,000/µL
Neutrophils	6,010	2,000-7,000/µL
Lymphocytes	2,020	1,000-4,000/µL
Hemoglobin	12.2	12-16 g/dL
Platelets	210,000	150,000-450,000/µL
Blood film	No abnormal cells seen	
Serum creatinine	39	70-110 µmol/L
Sodium	132.3	135-145 mmol/L
Potassium	4.5	3.5-4.5 mmol/L
Calcium	2.3	2.2-2.6 mmol/L
Magnesium	0.7	0.65-1.05 mmol/L
Aspartate aminotransferase	64.8	<40 U/L
Alanine aminotransferase	14.9	<35 U/L
Total protein	61.85	60-83g/L
Albumin	21.2	35-45 g/L
Globulin	40.65	30-40 g/L
Alkaline phosphatase	70	30-120 U/L
Total bilirubin	3.6	5-21 µmol/L
Direct bilirubin	2	1.71-20.5 µmol/L
PT/INR	1.01	0.8-1.1
Creatinine kinase	145.8	<171 µg/L
C-reactive protein	17	<6 mg/L
Erythrocyte sedimentation rate	110	mm/1^st^ hour
Thyroid-stimulating hormone	1.98	0.4-4 mIU/L
Random blood sugar	101	100-200 mg/dl
Cerebrospinal fluid red cells	Nil	
Cerebrospinal fluid lymphocytes	Nil	
Cerebrospinal fluid polymorphs	Nil	
Cerebrospinal fluid microscopy	Clear and colourless	
Cerebrospinal fluid protein	49.4	23-38mg/dl
Cerebrospinal fluid glucose	100.38	104.6 mg/dl
Cerebrospinal fluid culture	Negative	
Rheumatoid factor	Negative	
Antinuclear antibodies	Negative	
HIV 1 and 2 antigens/antibodies	Negative	
c-ANCA	Negative	
p-ANCA	Negative	
Serum cryoglobulin	Negative	
Hepatitis C antibody	Negative	
Hepatitis B surface antigen	Negative	
Urine full report	Normal. No active sediments	
Chest X-ray	Normal	
Ultrasound scan abdomen	Normal	
2D echocardiogram	Normal	
MRI brain and spine	Normal	
Renal and mesenteric angiogram	No features suggestive of polyarteritis nodosa or giant cell arteritis	

**Figure 3:**
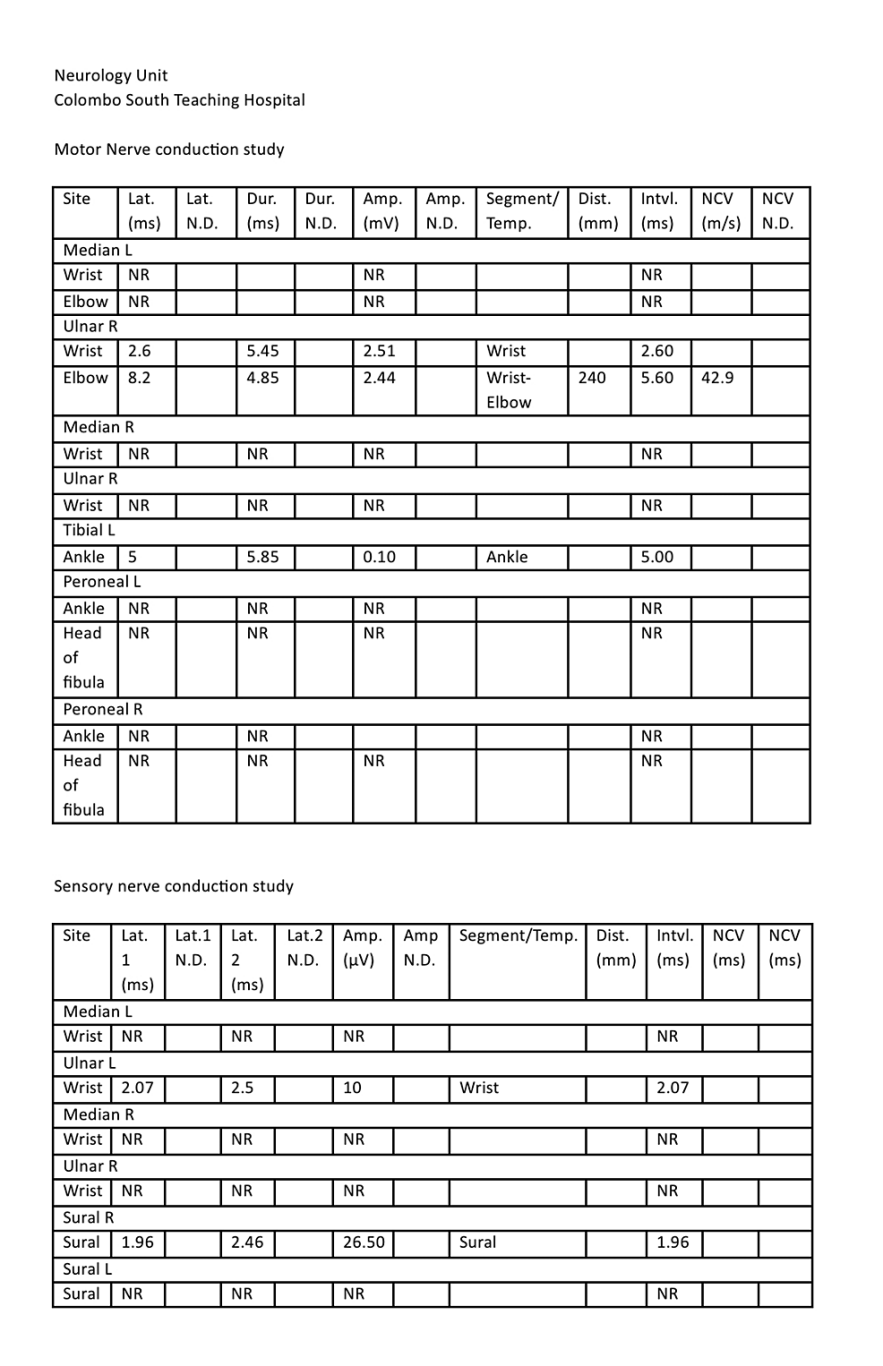
The second nerve conduction study Lat: latency; Dur: duration; Amp: amplitude; Temp: temperature; Dist: distance; Intvl: interval; NCV: nerve conduction velocity; ND: normal duration; L: left; R: right; NR: normal range

The NCS was repeated as there was a poor response even with five doses of IVIG (Figure 3). This revealed severe abnormal sensory and motor conduction, sparing the right side sural and ulnar nerves. The abnormal sensory and motor findings, along with marked electrodiagnostic asymmetry and vasculitic rash, were suggestive of acute fulminant vasculitic neuropathy (Table 2). A skin biopsy revealed leukocytoclastic vasculitis with a population of cells, predominantly neutrophils, distributed in perivascular and interstitial patterns around the vessels, giving rise to a busy dermis with superficial and mid-perivascular inflammation (Figure 2B). A left-side sural nerve biopsy was performed, but there were no features of vasculitis in the given sample.

**Table 2:** Results of the nerve conduction tests CMAP: compound motor action potential; AMAN: acute motor axonal neuropathy; GBS: Guillain-Barré syndrome

Nerve conduction test	Result
First nerve conduction test	The test shows normal sensory findings in the upper and lower limbs. The peripheral motor conduction is abnormal, with reduced CMAP amplitudes and conduction blocks. The velocities are normal. The electro-clinical findings are consistent with AMAN-type GBS.
Second nerve conduction test	This test shows severely abnormal sensory and motor conduction, sparing the right sural and right ulnar nerves. Previous normal sensory findings and motor conduction blocks were due to the early performance of the test before the Wallerian degeneration took place, mimicking axonal GBS.

Intravenous methylprednisolone 1 g/day was given for three days and continued with oral prednisolone 60 mg daily. Splints and orthotics were arranged along with physical therapy. The steroids were tapered off gradually after two weeks with the introduction of mycophenolate mofetil 500 mg twice daily.

The patient showed marked improvement with medical treatment and physiotherapy. The lower limb and upper limb power improved, and reflexes reappeared, however with persistent residual patchy sensory impairment. Furthermore, the vasculitic rash completely resolved.

## Discussion

The diagnosis of NSVN depends on the presence of clinical-histopathological features. Clinical diagnosis as per the Peripheral Nerve Society (PNS) guidelines requires typical features of vasculitic neuropathy, such as sensory or sensory-motor involvement with the asymmetrical distribution that mainly involves the lower limbs. Neuropathy is a distal predominant one with a characteristically painful, acutely relapsing course of a rapidly progressive nature. The gold standard of diagnosis for NSVN is a nerve biopsy with evidence of definite vasculitis. Although a nerve and muscle biopsy plays a pivotal role in the diagnosis of NSVN, a combined nerve and muscle biopsy is only performed if the muscle biopsy can be accessed through the same incision as the nerve. Despite the use of a full-thickness nerve biopsy, definitive histological evidence may still be elusive and enigmatic due to the patchy nature of the disease process, i.e., ‘skip lesions’ [5]. The sensitivity of the nerve and/or muscle biopsy for NSVN is estimated at 60%-70% at the most [5-7]. However, about 50% of patients with suspected vasculitic neuropathy lack histological examination [6]. Furthermore, adverse effects such as dysesthesia and persistent sensory loss may develop following a nerve biopsy [7]. In the face of these technical difficulties, the PNS Guidelines on NSVN suggest three levels of diagnosis of NSVN. In level 1, there is definitive vasculitic neuropathy, where all biopsy criteria are accompanied by vasculitic damage in the nerve biopsy. In level 2, there is clinical suspicion of vasculitic neuropathy, with some histopathological support, but it falls short of level 1. Level 3 describes probable cases with only clinical suspicion of vasculitic neuropathy in the absence of histological support [5,6,8,9].

**Figure 4:**
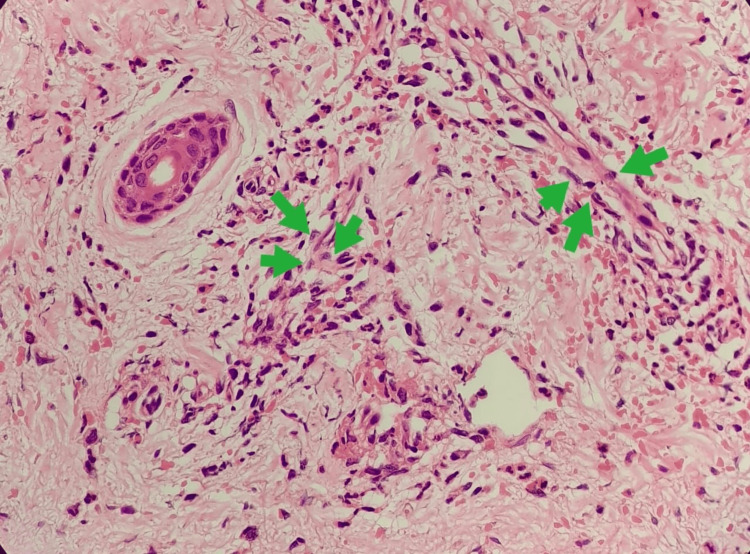
Six perivascular macrophages per vessel were seen on a 5 mm skin punch biopsy sample of the patient (green arrows).

A study conducted on biopsy-proven NSVN found that quantification of perivascular macrophages, with a cut-off of 2.7 macrophages per vessel on a 5 mm skin punch biopsy, had a sensitivity of 94% and a specificity of 79% for NSVN [8,10]. Our patient can be categorized as having level 3 clinical criteria with mononeuritis multiplex, vasculitic rash, severe neuropathic pain, and extensive investigations done to exclude other causes for vasculitic neuropathy [6,8]. These exclusions include conditions such as antineutrophil cytoplasmic antibodies (ANCA)-associated vasculitis, GBS, polyarteritis nodosa, cryoglobulinaemic vasculitis, secondary viral hepatitis, HIV-associated vasculitis, and metabolic causes such as diabetes and hypothyroidism. The perivascular macrophages on our patient's skin biopsy revealed that there are about six macrophages per vessel. As there are more than three macrophages per vessel shown in the above study on skin biopsy as a diagnostic tool for NSVN, it further confirms that our patient’s presentation can be attributed to NSVN (Figure 4) [8,10]. 

Non-systemic vasculitic neuropathy is caused by mononuclear inflammatory infiltrate, with or without necrosis, resulting in ischemic axonal degeneration [11]. Ischemic injury from the microvasculitic nerve appears to be the main etiopathogenetic event, along with immune-mediated mechanisms by complements, immunoglobulins, and significant immuno-expression of adhesion molecules (ICAM-1) in the neovascularization and endoneural microvessels, as well as tumor necrosis factor alpha (TNF-α) in the macrophages and Schwann cells, whereas interleukins are less significantly expressed [12].

Regardless of the categorization into subtypes, the damage caused during a vasculitic process determines the degeneration and subsequent necrosis of peripheral nervous structures, which are responsible for clinical symptoms [11]. In the specific case of NSVN, the presence of parietal damage to the vessels is needed, together with perivascular inflammation, to make the diagnosis of definite vasculitic neuropathy. It should be considered ‘probable’ as the involvement could be patchy otherwise. [11,12]

Vasculitic neuropathy can be a devastating condition if treatment is not initiated early and the diagnosis is missed. Our patient was treated for vasculitic neuropathy early, for which she achieved a near-complete recovery. It therefore demonstrates that even though the nerve biopsy should yield a negative result, vasculitic neuropathy is a possibility in the clinically suggestive presentation of NSVN, and a skin biopsy can also be used as an added diagnostic tool with good sensitivity and specificity. Furthermore, prompt treatment would indeed result in averting disabling defects that may affect the quality of life of patients [5,6].

The treatment of NSVN as per the 2010 Peripheral Nerve Society Guidelines Good Practice Point treatment recommendations is based on immunosuppression when considering the mechanism of immune-mediated vascular damage [13]. This requires corticosteroid monotherapy (1 mg/kg prednisolone, tapered to 10 mg daily at six months). This is preferred unless the neuropathy is rapidly progressive within four weeks. Combination therapy (corticosteroids with cyclophosphamide, methotrexate, mycophenolate mofetil, or azathioprine) should be used for patients as steroid-sparing agents [13]. We employed mycophenolate mofetil in our patient due to the lesser side effect profile and the drug availability. Rituximab is mainly used in systemic vasculitic neuropathy, like cryoglobulinaemic and ANCA-associated vasculitic neuropathy, or in severe NSVN [14].

## Conclusions

Non-systemic vasculitic neuropathy is an entity of vasculitic neuropathy that is under-recognized, under-evaluated, and easily misdiagnosed due to atypical presentations and wide clinical spectrum, especially due to negative nerve biopsy secondary to patchy involvement, like in our patient with significant clinical manifestations, positive nerve conduction test, and exclusion of all other causes for this presentation. If treated before progression to later stages with systemic involvement and complications, the outcome will be excellent and lifesaving. This case illustrates the importance of initial suspicion and a correct diagnosis of vasculitic neuropathy in the background of an atypical clinical picture for a near-complete cure of the disease. 
